# Meridional changes in the South Atlantic Subtropical Gyre during Heinrich Stadials

**DOI:** 10.1038/s41598-021-88817-0

**Published:** 2021-05-03

**Authors:** Tainã M. L. Pinho, Cristiano M. Chiessi, Rodrigo C. Portilho-Ramos, Marília C. Campos, Stefano Crivellari, Rodrigo A. Nascimento, Ana L. S. Albuquerque, André Bahr, Stefan Mulitza

**Affiliations:** 1 Institute of Geosciences, University of São Paulo, São Paulo, Brazil; 2 School of Arts, Sciences and Humanities, University of São Paulo, São Paulo, Brazil; 3 MARUM—Center for Marine Environmental Sciences, University of Bremen, Bremen, Germany; 4 Graduate Program in Geochemistry, Fluminense Federal University, Niterói, Brazil; 5 Institute of Earth Sciences, Heidelberg University, Heidelberg, Germany

**Keywords:** Palaeoceanography, Palaeoclimate

## Abstract

Subtropical ocean gyres play a key role in modulating the global climate system redistributing energy between low and high latitudes. A poleward displacement of the subtropical gyres has been observed over the last decades, but the lack of long-term monitoring data hinders an in-depth understanding of their dynamics. Paleoceanographic records offer the opportunity to identify meridional changes in the subtropical gyres and investigate their consequences to the climate system. Here we use the abundance of planktonic foraminiferal species *Globorotalia truncatulinodes* from a sediment core collected at the northernmost boundary of the South Atlantic Subtropical Gyre (SASG) together with a previously published record of the same species from the southernmost boundary of the SASG to reconstruct meridional fluctuations of the SASG over last ca. 70 kyr. Our findings indicate southward displacements of the SASG during Heinrich Stadials (HS) 6-4 and HS1, and a contraction of the SASG during HS3 and HS2. During HS6-4 and HS1, the SASG southward displacements likely boosted the transfer of heat to the Southern Ocean, ultimately strengthening deep-water upwelling and CO_2_ release to the atmosphere. We hypothesize that the ongoing SASG poleward displacement may further increase oceanic CO_2_ release.

## Introduction

Subtropical gyres are large systems of anticyclonic upper ocean circulation driven by wind stress curl^1, 2^, characterized as enormous reservoirs of heat and salt^3^. They are major pathways of energy redistribution between low and high latitudes with a pivotal role on the global climatic system^4^. As part of the subtropical gyres, western boundaries currents (e.g., Brazil Current (BC) and Gulf Stream) transport warm and salty tropical waters towards the poles, and eastern boundaries currents (e.g., Benguela and Canary Currents) transport cold and fresh waters towards the equator. At their midlatitude boundaries, subtropical gyres are limited by the Subtropical Fronts (STF).

Observational and model studies suggest a poleward migration of the subtropical gyres in the order of 0.1° per decade, driven by a systematic poleward displacement of the extratropical atmospheric circulation^5^. Changes in the geometry, strength and extension of the subtropical gyres may disturb the meridional heat transport with drastic consequences to marine ecosystems and the global climate system^5, 6^. An increased heat content in most subtropical western boundary currents has been registered during the last decades (Refs.^7, 8^). In the western South Atlantic, warming of the BC has been claimed to cause a severe dwindling of commercial fish stocks^9^. Even total marine productivity is projected to decline due to the expansion of the “ocean deserts”, i.e., the oligotrophic subtropical gyres^10, 11^. Yet, it is still not clear if modern changes in the subtropical gyres are promoted by anthropogenic activities or natural climate variability, mostly because of the relatively short instrumental observations. Paleoceanographic records offer a great opportunity to identify long-term changes in the subtropical gyres under different climatic conditions. This type of information is crucial to validate coupled climate models and improve our understanding of the potential anthropogenic role on the ongoing poleward shift of the subtropical gyres^5^.

Paleoceanographic studies provide evidences for latitudinal shifts of the Subtropical and the Subantarctic Fronts on orbital and millennial timescales (Refs.^12–16^). In the Atlantic sector of the Southern Ocean, the northward displacement of the Subtropical and the Subantarctic Fronts during full glacials hampered the exchange of heat and salt between the Atlantic and the Indian oceans through the Agulhas Leakage^12, 16^. In contrast, these fronts shifted southwards during millennial-scale climate events, e.g., Heinrich Stadials (HS)^13, 14^. Despite the knowledge about the Subtropical and Subantarctic Fronts, the past behavior of the South Atlantic Subtropical Gyre (SASG) and its consequences for the climate system remain unknown.

**Figure 1:**
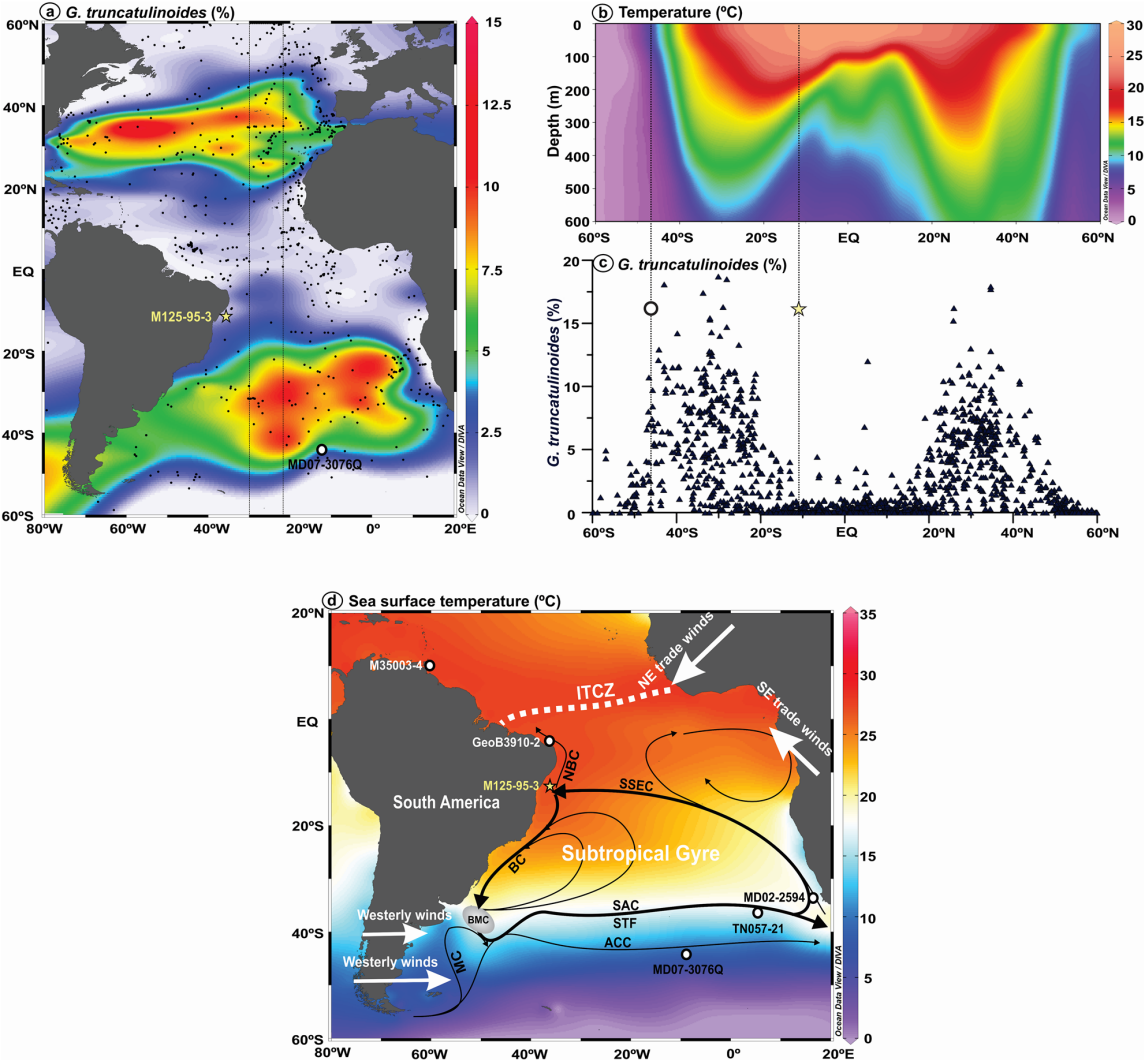
Location of marine sediment core M125-95-3 (yellow star) and other marine records discussed herein (open black dots). (**a**) Map of the modern relative abundance of planktonic foraminifera species *Globorotalia truncatulinoides* in the Atlantic Ocean^23^. Black dots represent the location of the surface sediment samples. The thin rectangle indicates the location of the temperature meridional profile depicted in panel “b”. (**b**) Mean annual temperature meridional profile for the upper 600 m of the water column of the Atlantic Ocean^25^. Vertical dotted lines delimit the South Atlantic Subtropical Gyre (SASG). See panel “a” for the location of the meridional profile. (**c**) Meridional profile of the modern relative abundance of *G. truncatulinoides* in the Atlantic Ocean^23^. The yellow star depicts the location of core M125-95-3 (this study) and the open black dot depicts the location of core MD07-3076Q^24^. (**d**) Mean annual sea surface temperature (color shading)^25^, schematic surface ocean circulation (black arrows)^26^ and atmospheric features (white arrows) of interest over the South Atlantic. Antarctic Circumpolar Current (ACC), Brazil Current (BC), Brazil–Malvinas Confluence (BMC), Intertropical Convergence Zone (ITCZ), Malvinas Current (MC), North Brazil Current (NBC), South Atlantic Current (SAC), Southern South Equatorial Current (SSEC), Subtropical Front (STF). The location of the following cores are depicted: M35003-4^27^, GeoB3910-2^28^, M125-95–3 (this study), MD02-2594^15^, TN057-21^14^, MD07-3076Q^24^. This figure was produced using the Ocean Data View software^29^ (ODV—version 5.2.1., https://odv.awi.de, 2020) and the CorelDRAW Graphics Suite software (CorelDRAW—version X6, https://www.coreldraw.com, 2012).

*Globorotalia truncatulinoides* is a deep-dwelling planktonic foraminiferal species that calcifies its shells within the permanent thermocline^17–19^. This species presents an one-year reproductive cycle with extensive vertical migration in the water column that is highly dependent on the stratification of the upper ocean^20, 21^. Therefore, *G. truncatulinoides* increases (decreases) in abundance when the thermocline is deeper (shallower)^19, 21^. The coiling direction of *G. truncatulinoides* has been commonly used to reconstruct the upper water column stratification^21, 22^, although surface sediments from the Atlantic Ocean reveal that the distribution of both sinistral and dextral morphotypes of *G. truncatulinoides* are closely related to the subtropical gyres^23^, showing high abundance inside the gyres, where the thermocline is deeper, and being virtually absent to the north and south of the gyres, where the thermocline is shallower (Fig. 1a–c). Thus, the abundance of *G. truncatulinoides* in adequately located marine sediment cores is an excellent proxy to track meridional changes in the subtropical gyres.

Here we compare the relative abundance of *G. truncatulinoides* from two sediment cores located on opposite sides of the modern SASG (Fig. 1), which are key locations to track past meridional displacements of the SASG^5^. We provide a new record of *G. truncatulinoides* abundance from core M125-95-3 (10.94° S, 36.20° W, 1897 m water depth) raised from the continental slope of the western tropical South Atlantic at the northern boundary of the SASG (nSASG) that covers the last ca. 70 kyr (Fig. 1a,d). We compare this record with the previously published abundance of *G. truncatulinoides* from core MD07-3076Q (44.92° S, 14.13° W, 3770 m water depth)^24^, collected at the southern boundary of the SASG (sSASG) (Fig. 1a,d). High temporal-resolution data from both cores (ca. 518 and 223 year, respectively, between adjacent samples) allow investigating millennial-scale changes in the SASG. The position of both cores has been strategically selected so that a meridional displacement of the SASG should cause antiphase excursions in *G. truncatulinoides* abundance in both cores. On the other hand, a contraction (expansion) of the SASG would cause a decrease (increase) in the abundance of *G. truncatulinoides* in both cores or a decrease (increase) in the abundance in one of the cores and no change in the other.

## Material and methods

Piston core M125-95–3 was collected from the continental slope of the western tropical South Atlantic during RV Meteor cruise M125 (Fig. 1a,d)^30^. We focus on the uppermost 7.4 m of the core which spans the last ca. 70 kyr covering all HS of the last glacial and deglacial periods (the age model was previously published in Ref.^31^). The age model is based on nine calibrated planktonic foraminifera accelerator mass spectrometry radiocarbon ages. For the portion of the core beyond the radiocarbon range, *Uvigerina* spp. stable oxygen isotopic (δ^18^O) tie-points were tuned to a benthic δ^18^O reference curve from Ref.^32^. The age modeling algorithm BACON v. 2.2^33^ was used within the software PaleoDataView v. 0.8.3.4^34^ to produce the age model. The relative abundance of planktonic foraminifera *G. truncatulinoides* were counted in the > 150 μm size fraction and its relative abundance was quantified from splits containing more than 300 specimens. We distinguished the sinistral and dextral morphotypes specimens of *G. truncatulinoides*, however, in the present study we choose to pool them together due to the very low abundance of the sinistral morphotype.

## Results

**Figure 2:**
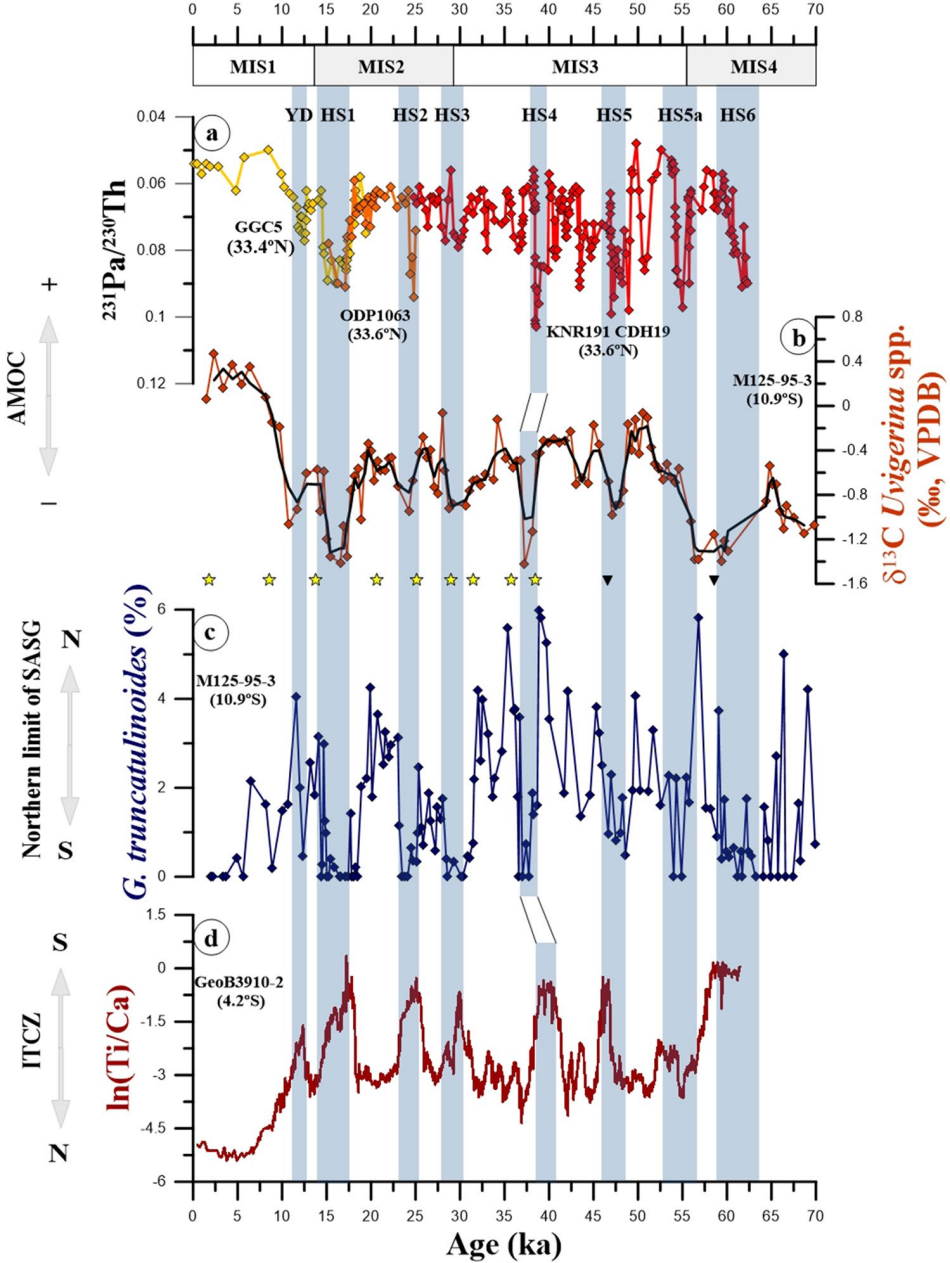
Comparison of relative abundance record of *Globorotalia truncatulinoides* from marine sediment core M125-95-3 with previously published records from the Atlantic Ocean. (**a**) ^231^Pa/^230^Th from the Bermuda Rise^35–37^. (**b**) *Uvigerina* spp. stable carbon isotopic composition (δ^13^C) from core M125-95-3 (running average of 3 points)^38^. (**c**) Relative abundance of *G. truncatulinoides* from core M125-95-3 (this study). (**d**) ln(Ti/Ca) from core GeoB3910-2 located in the northeastern Brazil^28^. Yellow stars on top of panel “c” depict calibrated radiocarbon ages and black triangles depict tie-points used to produce the age model of core M125-95-3 (2σ standard error smaller than symbol size)^31^. Blue vertical bars represent millennial-scale Heinrich Stadials (HS) 6-1 and the Younger Dryas (YD). Marine Isotope Stages (MIS) are depicted below the upper horizontal axis. Atlantic Meridional Overturning Circulation (AMOC), South Atlantic Subtropical Gyre (SASG), Intertropical Convergence Zone (ITCZ).

The relative abundance of *G. truncatulinoides* in core M125-95-3 ranges between 0 and 5.9%, with mean value of 1.7% during the last glacial period and 0.8% during the Holocene (Fig. 2c). Millennial-scale negative excursions of up to 5.9% characterize the relative abundance record of *G. truncatulinoides* and coincide with all HS of the last glacial and deglacial periods.

## Discussion

### Millennial-scale changes in the northern boundary of the South Atlantic Subtropical Gyre

The decreases in the relative abundance of *G. truncatulinoides* occurred simultaneously with increases in precipitation over NE Brazil that were, in turn, associated to southward displacements in the Intertropical Convergence Zone (ITCZ) during HS (Fig. 2c,d)^28, 39^. The meridional position of the ITCZ determines the location of the equatorial ascending branch of the Hadley Cells. The Hadley Cells from both hemispheres have an important role in the interhemispheric atmospheric heat transport^40^. Under a weak Atlantic Meridional Overturning Circulation (AMOC) (e.g., during HS) (Fig. 2a,b)^35–37^, the decreased northward oceanic heat transport warmed the South Atlantic^13, 41^. This resulted in a southward migration of the equatorial ascending branch of the Hadley Cells, partially compensating the decrease in northward oceanic heat transport via an increase in the northward atmospheric heat transport^42, 43^.

**Figure 3:**
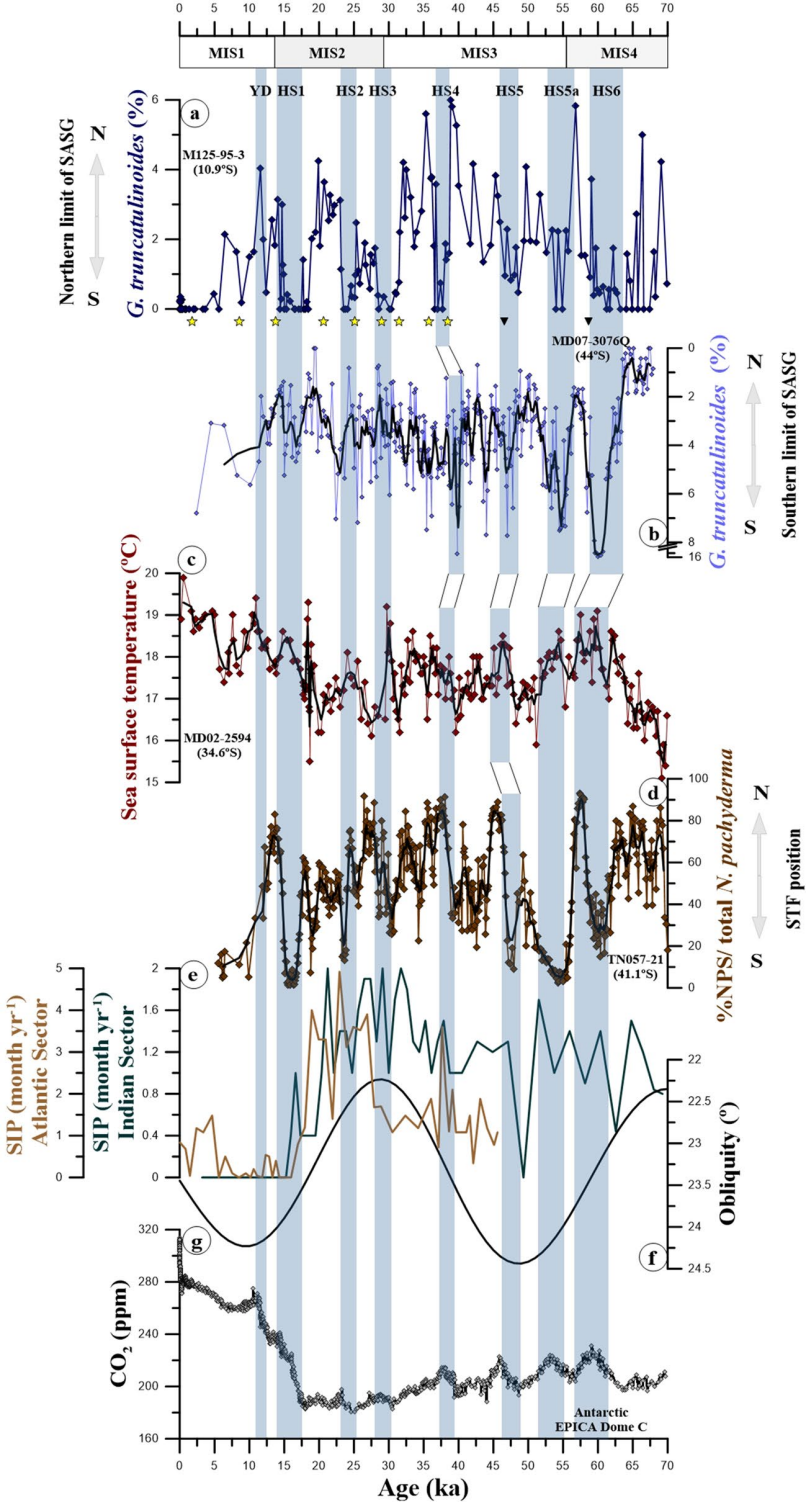
Relative abundance of *Globorotalia truncatulinoides* from cores M125-95-3 (this study) and MD07-3076Q^24^ located at the northern and southern limits of the South Atlantic Subtropical Gyre (i.e., nSASG and sSASG), respectively together with other proxy records discussed herein. (**a**) Relative abundance of *G. truncatulinoides* from core M125-95-3 (this study). (**b**) Relative abundance of *G. truncatulinoides* from core MD07-3076Q (running average of 5 points) (note the inverted axis)^24^. (**c**) Mg/Ca-based sea surface temperatures in the Agulhas Leakage (running average of 3 points)^15^. (**d**) Ratio of the percentage of *Neogloboquadrina pachyderma* (sinistral; NPS) to total *N. pachyderma* (sinistral and dextral) from core TN057-21 (running average of 7 points)^14^. (**e**) Southern Ocean sea-ice presence (SIP) in the Atlantic and Indian sectors of the Southern Ocean^52, 53^. (**f**) Obliquity^54^. (**g**) Atmospheric CO_2_ concentration^55^. Yellow stars on top of panel “b” depict calibrated radiocarbon ages and black triangles depict tie-points used to produce the age model of core M125-95–3 (2σ standard error smaller than symbol size)^31^. Blue vertical bars represent millennial-scale Heinrich Stadial (HS) 6 to 1 and the Younger Dryas (YD). Marine Isotope Stages (MIS) are depicted below the upper horizontal axis. Subtropical front (STF).

Changes in the Hadley Cells directly affect the oceanic Subtropical Cells (STC) by changing the trade winds stress on the surface. Indeed, the wind-driven oceanic STC can be described as the upper ocean counter-part of the Hadley Cells^44^. Therefore, changes in the meridional position of the equatorial branch of the STC are linked to the ITCZ position via the Hadley Cells^45^. During HS, McGee et al.^43^ described a southward shift of the ascending branch of the South Atlantic STC that followed the ITCZ^43^. The southward shift of the STC in the South Atlantic should be accompanied by a southward displacement of the nSASG during HS. We suggest that southward migrations of the nSASG during HS increased the upper water column stratification (i.e., shallower thermocline) at our core site (10.94° S, 36.20° W), decreasing the abundance of *G. truncatulinoides* (Figs. 2c, 3a). An increased stratification in the upper water column of the western tropical South Atlantic during HS has been confirmed by Portilho-Ramos et al.^46^ and Pedro et al.^47^ (Fig. S1d), supporting our suggestion.

In line with our results, in the tropical North Atlantic at the southern boundary of the North Atlantic Subtropical Gyre (sNASG), increases in the abundance of *G. truncatulinoides* (Fig. S1e)^27^ during HS suggest southward migrations of the sNASG. This is supported by upper water column temperature and salinity data^48^, as well as model experiments^49^, suggesting a tight coupling between the ascending branches of both STC and subtropical gyres in the Atlantic during HS.

The suggested southward migrations of the nSASG during HS were also accompanied by decreases in the strength of the SE trade winds^50^, that, in turn, are a consequence of the decreased meridional sea-surface temperature (SST) gradient in the tropical South Atlantic^13^. The reduced strength of the SE trade winds was thus co-responsible for the increases in upper water column stratification in the western tropical South Atlantic. At our core site, however, the large amplitude decreases in the abundance of *G. truncatulinoides* (Figs. 2c, 3a) point to the occurrence of changes in upper water column structure. Such changes would be accomplished by the nSASG crossing southwards our core site (Fig. 1).

Moreover, our core site is located at the modern bifurcation of the South Equatorial Current (SEC) in the upper 100 m of the water column (Fig. 1d). Our suggestion of southward migrations of the nSASG to be dynamically linked to southward shifts of the ITCZ position during HS contrasts to the seasonal mode of changes in the SEC bifurcation (i.e., during austral summer, a southward migration of the ITCZ occurs simultaneously to a northward migration of the SEC bifurcation)^51^. Thus, our results highlight the need to consider the timescale while investigating the processes responsible for changes in western tropical South Atlantic upper water column stratification.

### Impacts of changes in the south Atlantic subtropical gyre

The *G. truncatulinoides* abundance records from the nSASG (core M125-95-3) and the sSASG (core MD07-3076Q) reveal an antiphase pattern during HS6-4 and HS1 (Fig. 3a,b). Notably, in both *G. truncatulinoides* records sinistral and dextral morphotypes were quantified together. While in the nSASG *G. truncatulinoides* abundance decrease during HS6-1 (Fig. 3a), in the sSASG *G. truncatulinodes* abundance increase during HS6-4 and HS1 with no clear trend during HS3 and HS2 (Fig. 3b)^24^. The antiphase pattern suggests that the whole SASG was displaced southwards during HS6-4 and HS1. In contrast, the reduction in *G. truncatulinoides* in the nSASG together with constant values in the sSASG suggest a meridional contraction of the SASG during HS3 and HS2 (Fig. 3a,b).

### Southward migration of the South Atlantic Subtropical Gyre during Heinrich Stadials 6–4 and 1

The Southern Hemisphere westerly winds control the position of the STF in the South Atlantic (e.g., Ref.^56^). A southward displacement of the STF during HS has been suggested^57^. The concurrent HS increases in the abundance of *G. truncatulinoides* in the sSASG (Fig. 3b) and the decreases in dust flux around Antarctica (a proxy for the Southern Hemisphere westerly winds intensity) suggest a link between the southward displacements of the sSASG and the Southern Hemisphere westerly winds^58^. The southward (northward) displacement of the STF has commonly been correlated to the increased (reduced) water inflow from the Indian to the Atlantic Ocean through the Agulhas Leakage^59, 60^. A SST record under the influence of the Agulhas Leakage indeed shows systematic millennial-scale increases during HS, indicating southward shifts of the STF (Fig. 3c)^15^. Also, a planktonic foraminiferal index for the relative position of the STF in the South Atlantic (% *Neogloboquadrina pachyderma* (sinistral) / *N. pachyderma* total) corroborates the southward migrations of the STF during HS (Fig. 3d)^14^. The strong correlation between the *G. truncatulinoides* record from the sSASG, Agulhas Leakage SST and the STF index (Fig. 3b–d) suggest that the position of the sSASG was closely related to the Southern Hemisphere westerly winds and the STF. We suggest that the extratropical atmospheric circulation accompanied the southward displacement of the ITCZ and the nSASG during the HS6-4 and HS1, as indicated by model experiments^43^.

Model simulations of a collapsed AMOC show a positive temperature anomaly (ca. 4 °C) at ca. 500 m water depth in the SASG and a deepening in the thermocline in the sSASG^47^, indications of an increase in the heat content of the SASG and a southward shift in the sSASG, respectively. Concurrently, in the Antarctic Circumpolar Current the increased eddy heat transport together with a southern position of the westerlies, likely allowed for more heat to reach high southern latitudes causing a retreat in Antarctic sea ice^47, 61^. The southward displacement of the sSASG probably produced a steeper meridional SST gradient in the mid-latitudes of the South Atlantic. This steeper gradient may have contributed to stronger and southward-shifted westerlies, strengthening Southern Ocean deep-water upwelling^62^. Increased upwelling around Antarctica, in turn, fostered CO_2_ release to the atmosphere^63^. A weakened dust-driven biological pump in the Southern Ocean also contributed to the rise in atmospheric CO_2_ during HS (e.g., Ref.^64^) (Fig. 3g).

### Contraction of the South Atlantic Subtropical Gyre during Heinrich Stadials 3–2

Decreases in *G. truncatulinoides* abundance at the nSASG and the absence of major changes in the abundance of this species at the sSASG (Fig. 3a,b) suggest a meridional contraction of the SASG during HS3 and HS2. At the end of Marine Isotope Stage (MIS) 3 and during most of MIS2, the abundance of *G. truncatulinoides* at the sSASG^24^ shows nearly constant values between 2–4% (Fig. 3b). This is the period (i.e., ca. 30–19 ka) when full glacial boundary conditions (e.g., lowest seal level, largest sea ice expansion, and lowest atmospheric CO_2_ concentration) were reached. This period encompasses HS3 and HS2, which were not related to southward shifts of the sSASG (Fig. 3b). We suggest that the full glacial boundary conditions hindered the sSASG to migrate southwards even under HS forcing. Under full glacial boundary conditions, the northward migration of the Polar and Subantarctic Fronts together with extensive sea-ice around Antarctica probably hampered southward displacements of the sSASG. The striking increase in sea-ice in the Atlantic and Indian sectors of the Southern Ocean under full glacial boundary conditions corroborates this suggestion (Fig. 3e)^52, 53^. The long-term expansion of sea-ice equatorwards was fostered by low obliquity^65^ that reached minimum value at ca. 30 ka (Fig. 3f)^54^. Changes in sea-ice extent should be accompanied by changes in the oceanic Polar and Subantarctic Fronts (e.g., Refs.^12, 16, 66, 67^). Records on millennial-scale temporal resolution of the Agulhas Leakage SST, the position of the STF and dust flux to the Southern Ocean (Fig. 3c,d)^14, 15, 58^ confirm the presence of full glacial boundary conditions during HS3 and HS2. Importantly, full glacial boundary conditions were associated to a significant northward displacement of the Southern Hemisphere westerly winds and a marked decrease in Southern Ocean deep-water upwelling, that hindered CO_2_ to be released from the Southern Ocean to the atmosphere, as recorded in ice-cores during HS3 and HS2 (Fig. 3e–g)^55^.

**Figure 4:**
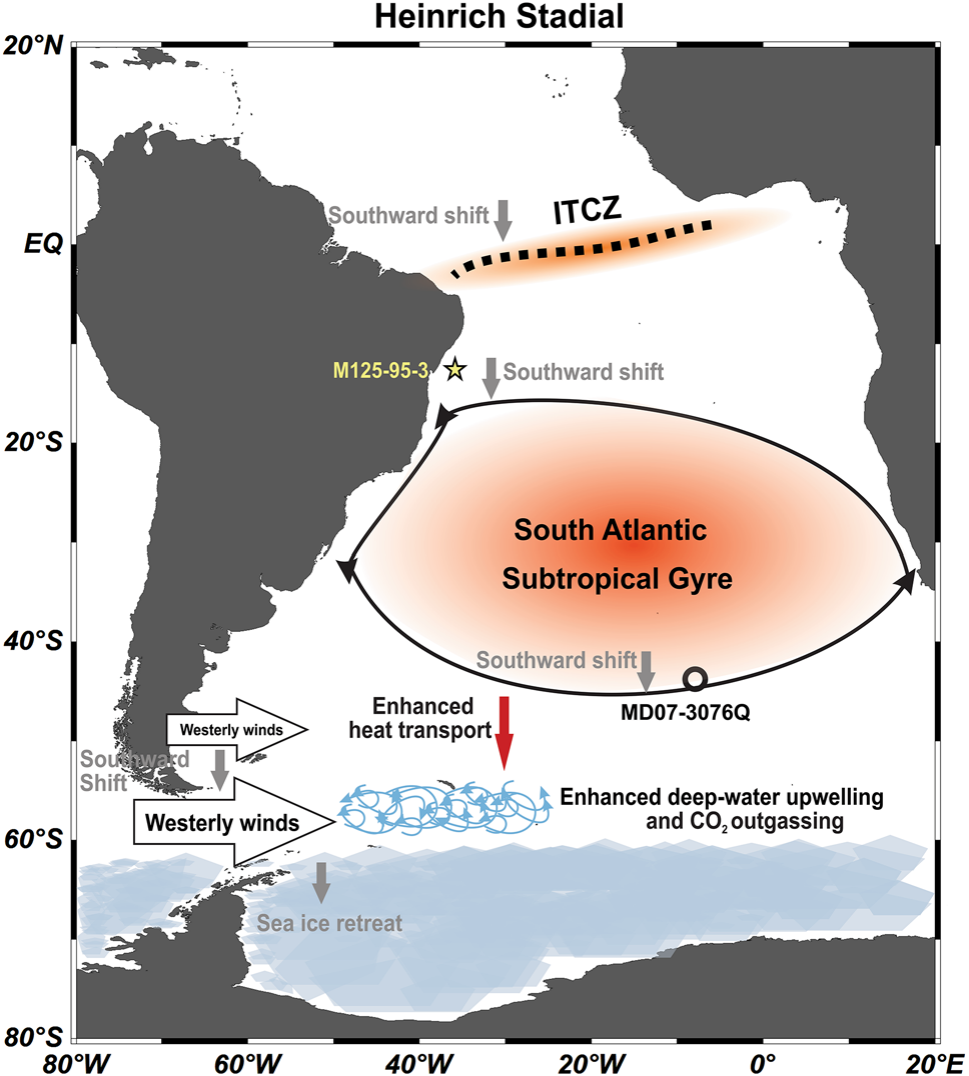
Schematic representation of the mechanism by which a southward displacement of the South Atlantic Subtropical Gyre during a Heinrich Stadial transfer more heat to the Southern Ocean, ultimately fostering CO_2_ release to the atmosphere. This figure was produced using the Ocean Data View software^29^ (ODV—version 5.2.1., https://odv.awi.de, 2020) and the CorelDRAW Graphics Suite software (CorelDRAW—version X6, https://www.coreldraw.com, 2012).

In summary, enhanced poleward heat fluxes occurred during HS6-4 and HS1 and were favored by southward shifts of the SASG (Fig. 4). Such meridional migrations of the SASG may have played a central role on oceanic carbon storage or release during the last glacial period on millennial timescales by controlling heat delivery to the Southern Ocean (Fig. 4). Regarding the ongoing poleward displacement of the SASG^5^, our results suggest that an increase in heat transport to the Southern Ocean may strengthen deep-water upwelling and CO_2_ release to the atmosphere, constituting a positive feedback for global warming.

## Conclusions

Here we present the abundance of *G. truncatulinoides* as a new proxy for the meridional displacement of the SASG on millennial timescales. Our *G. truncatulinoides* abundance record, together with previously published records, show that the SASG migrated southwards during most HS of the last glacial and deglacial periods (i.e., HS6-4 and HS1). These events were probably responsible for the transfer of substantial amounts of heat from the SASG to the Southern Ocean, ultimately strengthening deep-water upwelling and CO_2_ release to the atmosphere. On the other hand, the SASG contracted during HS3 and HS2, likely resulting in decreased poleward heat transfer, deep-water upwelling and CO_2_ release to the atmosphere. The contraction was forced by full glacial boundary conditions, namely equatorwards advance in sea-ice as well as in the position of the Polar and Subantarctic Fronts. While several studies previously described the release of CO_2_ to the atmosphere during millennial-scale stadials, our novel mechanism suggests that the poleward heat transfer from the SASG to the Southern Ocean had a pivotal role in this process. Our results indicate that the ongoing poleward displacement of the SASG may drive oceanic CO_2_ release that will act as a positive feedback to global warming.

## Material and methods

### Modern distribution of Globorotalia truncatulinoides

The modern spatial distribution of planktonic foraminifera *Globorotalia truncatulinoides* in Atlantic Ocean sediments was extracted from *Kucera *et al.^23^. The comparison of its spatial distribution with upper water column structure, circulation and physico-chemical properties suggests that *G. truncatulinoides* track the meridional position of the South Atlantic Subtropical Gyre^19, 21, 23^. *Globorotalia truncatulinoides* shows high abundance inside the gyre where the thermocline is deep, and is virtually absent to the north and south of the gyre where the thermocline is shallow. In *Kucera *et al.^23^, foraminifera were picked from the > 150 μm size fraction of sample splits containing around 300 specimens^23^, the same method applied here. Further details on the ages of modern surface sediments can be found in *Kucera *et al.^23^. Modern foraminiferal data used here are available from the World Data Center PANGAEA (https://doi.pangaea.de/10.1594/PANGAEA.841194).

### Marine sediment core

Marine sediment core M125-95–3 (10.94° S, 36.20° W, 1897 m water depth, 1040 cm long) was collected from the continental slope of the western tropical South Atlantic during RV Meteor cruise M125^30^. Here we examined the upper 740 cm of the core that span the last ca. 70 kyr and cover all Heinrich Stadials (HS) of the last glacial and deglacial periods. This section was sampled with 10 cm^3^ syringes. Samples were wet-sieved, oven-dried at 50 °C and the size fraction higher than 125 µm was stored in glass vials. Faunal analyses were conducted every 10 cm for the whole investigated section but sampling space was decreased in the neighborhood and within every HS. 144 samples were analyzed.

### Identification of planktonic foraminifera

The wet-sieved and oven-dried > 125 μm fraction was dry-sieved in a 150 μm sieve, and the > 150 μm fraction was used for the determination of the relative abundances of the species that were quantified from splits containing more than 300 specimens. Taxonomy was based on *Stainforth *et al.^68^ and *Hemleben *et al.^69^). Here we present the abundance of planktonic foraminifera species *Globorotalia truncatulinoides* (both dextral and sinistral morphotypes). Since sediment core M125-95–3 was collected well above the glacial lysocline depth^70^, we considered the effect of dissolution in our planktonic foraminiferal faunal composition to be negligible. The planktonic foraminifera *G. truncatulinoides* record from sediment core MD07-3076Q was previously published by *Gottschalk *et al.^24^.

### Age model

The age model of sediment core M125-95-3 was previously published by *Campos *et al.^31^. It combines nine calibrated planktonic foraminifera accelerator mass spectrometry radiocarbon ages and tunning benthic foraminifera δ^18^O tie-points to a benthic δ^18^O reference curve from *Govin *et al.^32^. The age modeling algorithm BACON v. 2.2^33^ was used within the software PaleoDataView v. 0.8.3.4^34^ for age-depth modeling.

## Supplementary Information

Supplementary Information
