# Supplementary material for: Meridional changes in the South Atlantic Subtropical Gyre during Heinrich Stadials
Source: Sci Rep. 2021 May 3;11:9419. doi: 10.1038/s41598-021-88817-0 (PMC8093259; doi:10.1038/s41598-021-88817-0)
Supplement: Supplementary file 1 — Supplementary Information [file 41598_2021_88817_MOESM1_ESM.pdf]

Supplementary Information

Meridional changes in the South Atlantic Subtropical Gyre during Heinrich Stadials

Tainã M. L. Pinho[1*]; Cristiano M. Chiessi[2]; Rodrigo C. Portilho-Ramos[3]; Marília C. Campos[1]; Stefano Crivellari[2]; Rodrigo A. Nascimento[4]; André Bahr[5]; Ana L.S. Albuquerque[4]; Stefan Mulitza[3]

[1] Institute of Geosciences, University of São Paulo, São Paulo, Brazil

[2] School of Arts, Sciences and Humanities, University of São Paulo, São Paulo, Brazil

[3] MARUM – Center for Marine Environmental Sciences, University of Bremen, Bremen, Germany

[4] Graduate Program in Geochemistry, Fluminense Federal University, Niterói, Brazil

[5] Institute of Earth Sciences, Heidelberg University, Heidelberg, Germany

* Correspondence and requests for materials should be addressed to T.M.L.P (email: taina.pinho@usp.br)

Figure S1:

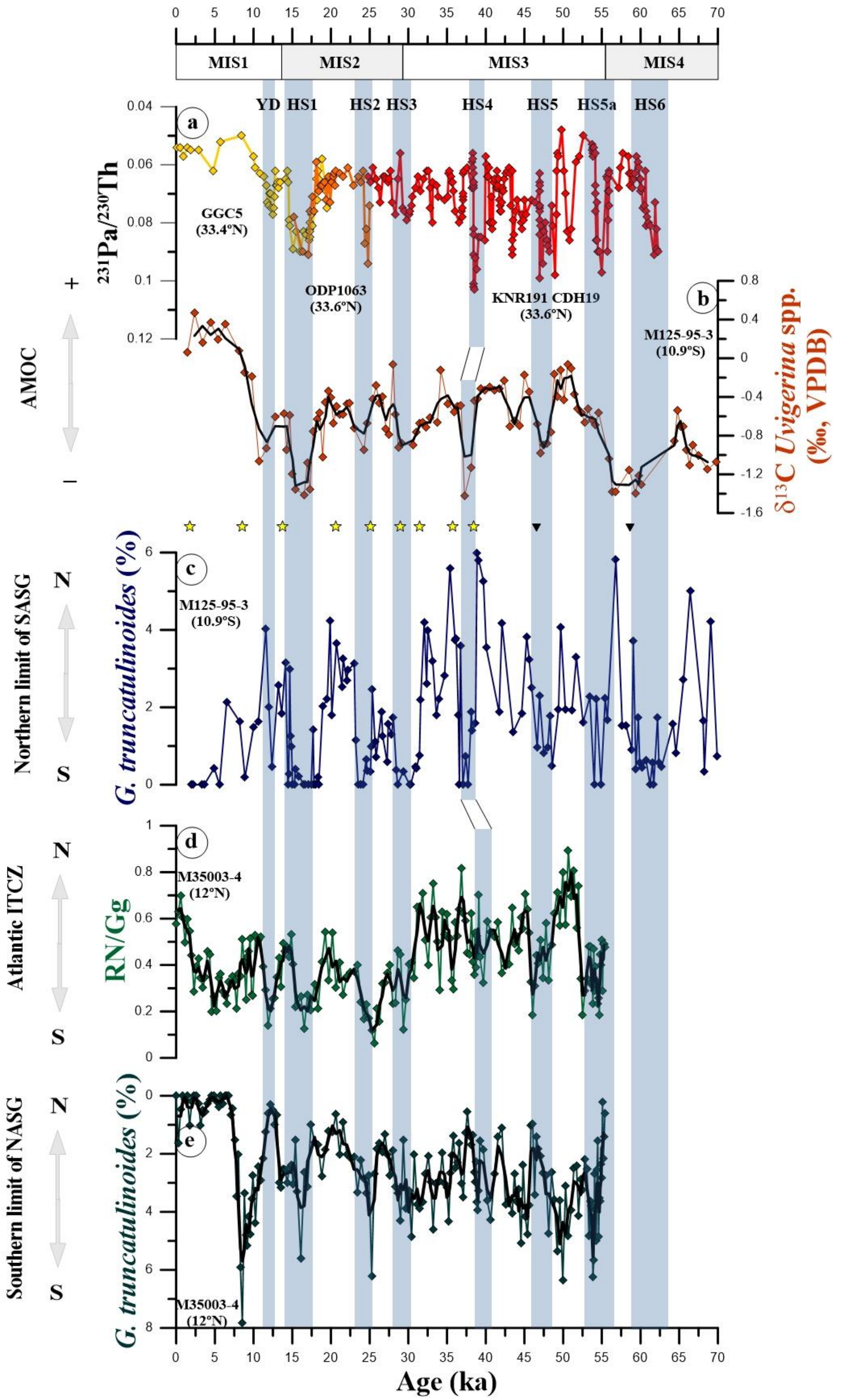

MIS1
MIS2
MIS3
MIS4
YD
HS1
HS2
HS3
HS4
HS5
HS5a
HS6
a
^{231}Pa/^{230}Th
GGC5
(33.4°N)
ODP1063
(33.6°N)
KNR191 CDH19
(33.6°N)
b
M125-95-3
(10.9°S)
δ^{13}C *Uvigerina* spp.
(‰, VPDB)
AMOC
+
–
c
M125-95-3
(10.9°S)
G. truncatulinoides (%)
Northern limit of SASG
N
S
d
M35003-4
(12°N)
RN/Gg
Atlantic ITCZ
N
S
e
M35003-4
(12°N)
G. truncatulinoides (%)
Southern limit of NASG
N
S
Age (ka)

Supplementary Figure Captions

Figure S1. Relationship between the Atlantic Intertropical Convergence Zone and northern limit of the South Atlantic Subtropical Gyre (nSASG). **(a)** Bermuda Rise ^{231}Pa/^{230}Th[1–3]; **(b)** δ^{13}C *Uvigerina* spp. from the study site[4] **(c)** Relative abundance of *Globorotalia truncatulinoides* from core M125-95-3; **(d)** %*Neogloboquadrina*/(%*Neogloboquadrina* + %*G. glutinata*) (RN/Gg) ratio as a proxy for the position of the Atlantic ITCZ in the Tobago Basin core M35003–4[5]; **(e)** Relative abundance of *Globorotalia truncatulinoides* from core M35003-4 (Tobago Basin)[6]. Yellow stars on top of panel "c" depict calibrated radiocarbon ages and black triangles depict tie-points used to produce the age model of core M125-95-3 (2σ standard error smaller than symbol size)[7]. Blue vertical bars represent millennial-scale Heinrich Stadials (HS) 6-1 and the Younger Dryas (YD). Marine Isotope Stages (MIS) are depicted below the upper horizontal axis. Atlantic Meridional Overturning Circulation (AMOC), Intertropical Convergence Zone (ITCZ), North Atlantic Subtropical Gyre (NASG), South Atlantic Subtropical Gyre (SASG).

Supplemantary References

1. McManus, J. F., Francois, R., Gherardl, J. M., Kelgwin, L. & Drown-Leger, S. Collapse and rapid resumption of Atlantic meridional circulation linked to deglacial climate changes. *Nature* **428**, 834–837 (2004).
2. Lippold, J. *et al.* Does sedimentary 231Pa/230Th from the Bermuda Rise monitor past Atlantic Meridional Overturning Circulation? *Geophys. Res. Lett.* **36**, 1–6 (2009).
3. Henry, L. G. *et al.* North Atlantic ocean circulation and abrupt climate change during the last glaciation. *Science (80-.).* **353**, 470–474 (2016).
4. Campos, M. C. *et al.* Constraining Millennial-Scale Changes in Northern Component Water Ventilation in the Western Tropical South Atlantic. *Paleoceanogr. Paleoclimatology* **35**, 1–32 (2020).
5. Portilho-Ramos, R. C. *et al.* Coupling of equatorial Atlantic surface stratification to glacial shifts in the tropical rainbelt. *Sci. Rep.* **7**, (2017).
6. Hüls, M., & Zahn, R. Millennial-scale sea surface temperature variability in the western tropical North Atlantic from planktonic foraminiferal census counts. *Paleoceanography* **15**, 659–678 (200AD).
7. Campos, M. C. *et al.* A new mechanism for millennial scale positive precipitation anomalies over tropical South America. *Quat. Sci. Rev.* **225**, (2019).
